# The Path From Awareness to Action: Exploring Diabetic Patients' Awareness and Attitudes and Barriers to Utilization of Artificial Pancreas in the Beheira Governorate, Egypt

**DOI:** 10.7759/cureus.52703

**Published:** 2024-01-22

**Authors:** Sameer H Hafez, Noha A Mohammed, Abeer Yahia Mahdy Shalby, Elsadig Eltaher Hamed Abdulrahman, Ahmed Farhan AlQarni, Fahad Ayed Alhamami, Hashem Fahd Alshehri, Mohammed Khalid Hussein, Mohamed Mustafa Abd Alganny, Mohamed Saied Harfoush

**Affiliations:** 1 Community Health Nursing, Beni-Suef University, Beni-Suef, EGY; 2 Community and Mental Health Nursing, Najran University, Najran, SAU; 3 Medical and Surgical Nursing, Benha University, Benha, EGY; 4 Faculty of Nursing, College of Nursing, Najran University, Najran, SAU; 5 Medical and Surgical Nursing, Faculty of Nursing, Najran University, Najran, SAU; 6 Histopathology Laboratory, Najran Armed Forces Hospital, Najran, SAU; 7 Laboratory Quality Management, Najran Armed Forces Hospital, Najran, SAU; 8 Hematology, Najran Armed Forces Hospital, Najran, SAU; 9 Medical and Surgical Nursing, Faculty of Nursing, Jazan University, Jazan, SAU; 10 Community Health Nursing, Faculty of Nursing, Mansoura University, Mansoura, EGY; 11 Nursing, College of Applied Medical Sciences, Buraydah College, Qassim, SAU; 12 Community Health Nursing, Faculty of Nursing, Damanhour University, Damanhour, EGY

**Keywords:** attitude, utilization barriers, knowledge, artificial pancreas, diabetic patients

## Abstract

Background and objective

There is scarce data on diabetic patients’ awareness, attitude, and barriers to utilization regarding the artificial pancreas. In light of this, the current study aimed to explore the awareness, attitudes, and perceived barriers to utilization of the artificial pancreas experienced by diabetic patients.

Methods

A cross-sectional study design was employed to achieve the aim of the study. The study was conducted in Damanhur city, the Beheira Governorate, Egypt. The convenience sampling technique was used to include 385 diabetic patients. The researchers designed an interview questionnaire comprising four parts to collect data about knowledge, attitudes, and barriers to utilization.

Results

The findings showed that 61% of the participants had a satisfactory level of overall knowledge. Regarding overall attitude, 64.1% of participants exhibited a positive attitude toward the artificial pancreas. The data indicated that 37.7% of participants identified the associated high cost as a significant barrier. Additionally, 23.3% expressed concerns about the lack of healthcare provider support, 21.5% had reservations regarding maintenance, and 17.5% felt limited by their technical skills.

Conclusions

The study revealed a notable satisfactory level of knowledge and attitudes among about two-thirds of participants regarding the artificial pancreas. Concerns about the high cost emerged as a predominant barrier followed by a lack of healthcare provider support. Empowering both healthcare providers and patients through ongoing educational initiatives can play a pivotal role in fostering a positive attitude and addressing concerns related to artificial pancreas technology.

## Introduction

About 422 million people worldwide are affected by diabetes, with the majority of them living in low- and middle-income countries such as Egypt. Every year, 1.5 million deaths are directly attributed to diabetes. In recent decades, there has been a steady increase in the prevalence of diabetes [1]. The Gulf Cooperation Council (GCC) countries, including Saudi Arabia, Bahrain, Qatar, Oman, Kuwait, and the United Arab Emirates, are among the regions with the highest prevalence of diabetes. These countries share similar population characteristics, such as religion, language, lifestyle, diet, and income. The prevalence of diabetes in GCC ranges from 8 to 22%, according to the latest report from the International Diabetes Federation (IDF) [2]. Despite remarkable advances in the treatment of type 1 diabetes, the achievement of optimal glycemic control without the occurrence of hypoglycemia remains a challenge for patients of all ages as well as healthcare providers. The current approach to insulin therapy for type 1 diabetes involves either injecting insulin multiple times a day or infusing insulin continuously under the skin using an artificial pancreatic insulin pump [3,4].

Artificial pancreas therapy is an emerging treatment option that combines an insulin pump and a continuous glucose monitor with a control algorithm to deliver insulin in a glucose-responsive manner (i.e., a single-hormone artificial pancreas system). Glucagon can also be delivered in a similar glucose-responsive manner, as in dual-hormone systems. Therefore, the use of an artificial pancreas can reduce the burden on the patient by automatically adjusting the amount of insulin entering the body based on sensor glucose levels. Several artificial pancreas systems are currently in development and their safety and effectiveness are being assessed in many studies, with promising results so far [5].

Artificial pancreas systems significantly reduce the daily burden of decision-making. With minimal user effort, these systems optimize the management of blood glucose levels, thereby increasing the amount of time spent in the target range. For example, in the event of a hypoglycemic episode during sleep, the hybrid closed-loop technology will temporarily suspend insulin delivery to minimize the time spent below the target range. In addition, if the sensor anticipates a potential hypoglycemic event, insulin delivery is reduced or temporarily suspended. If the sensor detects that hyperglycemia is imminent, the system may temporarily increase the basal insulin rate or deliver a small correction bolus, or execute a combination of both. Bimodal insulin and glucagon regimens are designed to mimic natural pancreatic function and can provide tightly controlled blood glucose without causing hypoglycemia. Current research is investigating the effectiveness of different combinations of hormones in controlling glycemic levels [6,7].

Artificial pancreas systems demand continuous user engagement and are not entirely hands-free. Consistent upkeep is vital to ensure proper functionality, involving tasks like inputting meal sizes and verifying the placement and replacement of continuous glucose monitoring (CGM) and infusion pump catheters. Regular assessments of CGM accuracy and sensor replacement are necessary. Users are required to manually input meal carbohydrate amounts and adjust computer program settings for precise insulin delivery, aiming to keep blood glucose within the target range. Troubleshooting may involve restarting or reconnecting the CGM, infusion pump, and computer program. Additionally, users may need to address high or low blood glucose levels if the system struggles to maintain the desired range. The use of adhesive patches may lead to redness or irritation, and certain medications may interfere with glucose monitoring [8].

Currently, the artificial pancreas system is the most advanced technology to help reduce the risk of exercise-induced hypoglycemia. The current study aimed to explore the awareness, attitudes, and perceived barriers to utilization regarding the artificial pancreas experienced by diabetic patients. The current study addressed three research questions to achieve the objective of the study: (1) What is the level of awareness among diabetic patients regarding artificial pancreas technology? (2) What are the prevailing attitudes of diabetic patients towards the use of artificial pancreas technology in diabetes management? (3) What are the perceived barriers hindering the use of artificial pancreas technology among diabetic patients?

## Materials and methods

Study design and sample

A cross-sectional study design was employed in this study. This design enables the concurrent gathering of data from a varied sample of participants at a specific moment, thereby providing a snapshot of the current perspectives within the population. The study was conducted at outpatient clinics, primary healthcare centers, and community settings in Damanhur city, the Beheira Governorate, Egypt. A convenience sampling method was used to enroll the participants The sample size was determined through a systematic calculation using the below formula: 

Sample size = (1.962) x (50) x (1-0.5) / 0.052.

This approach takes into account various factors, including the target confidence level (set at 1.96 for a 95% confidence interval), the margin of error (0.052), and the estimated proportion of the population exhibiting a specific characteristic (in this case, 50%). By applying this method, the calculated sample size was 385.

Inclusion and exclusion criteria

The eligibility criteria for this study included individuals diagnosed with type 1 diabetes, aged 18 years and older, possessing sufficient cognitive ability to understand and respond to questions related to the artificial pancreas, and expressing a willingness to provide informed consent for their participation in the research study. Individuals who did not meet the specified inclusion criteria were excluded.

Data collection

The researcher designed the interview questionnaire comprising four parts. Part 1 was dedicated to obtaining socio-demographics and history of the disease, and included factors such as age, gender, educational level and residence, and the duration from disease onset. The second part focused on assessing the knowledge of the studied sample about artificial pancreas, and it comprised nine questions that covered various aspects. This part of the questionnaire drew upon existing literature, incorporating insights from a few studies [8,9]. Each question was scored from 1 to 3, as follows: 1: don’t know/wrong answer, 2: an incomplete answer, and 3: a complete answer. The total score was summed up and classified as satisfactory if it was >50% and unsatisfactory if ≤50%. The third part, centered on attitudes regarding the use of the artificial pancreas, incorporated six questions derived from various studies [10-12]. Each item was scored 1 if the answer was "not sure", 2 if "maybe", and 3 if "sure". The total score of this part was 18. The total score was summed up and categorized into positive attitude if the percentage was ≥60% and negative attitude if it was <60%. The last part involved an open-ended question about the most common utilization barrier.

The validation of the questionnaire was performed by rigorously assessing to ensure the robustness of the data collection instruments. A panel of five professors specializing in community health nursing undertook the validation process, bringing diverse expertise to the evaluation. The consistency, as measured by Cronbach's Alpha coefficient test, was 0.78 for the knowledge section, and 0.83 for the attitude section. A pilot study to evaluate the content of the questionnaire and the time required for data collection was conducted among 40 diabetic patients. Patients in the pilot were excluded from the final analysis.

The data collection phase extended from January 2023 to September 2023, providing a substantial timeframe to comprehensively achieve a snapshot of the awareness and acceptance of artificial pancreas. The multi-month data collection window aligns with the meticulous approach of the study, contributing to a nuanced understanding of the dynamics surrounding the artificial pancreas. The researchers met the participants at outpatient clinics, primary healthcare centers, and community settings. The objective of the study was explained at the outset and oral consent was obtained. The data was collected by interviewing all the participants.

Ethical considerations

The approval for the study was obtained from the Research Ethics Committee of the Faculty of Nursing at Damanhur University on 17/11/2022 under code number (2022-65-d). The research was conducted in a scrupulously ethical manner, prioritizing the welfare and rights of the participants. Before the commencement of data collection, participants received a thorough explanation of the study objectives, procedures, and potential implications. Oral informed consent was obtained from each participant, signifying their voluntary agreement to participate in the study. To protect the confidentiality and privacy of participants, a strict data management protocol was implemented. All data collected were treated in the strictest confidence and used only for research purposes. To maintain the anonymity of participants in public reports, individual names were replaced with code numbers during data analysis and reporting.

Statistical analysis

The thorough examination of research participants involved a careful exploration of diverse aspects, encompassing demographics as well as knowledge and attitudes about the artificial pancreas. Descriptive statistics formed the foundation of this exploration, providing a detailed portrayal of the study cohort. SPSS Statistics v. 24.0 served as the analytical engine, aiding in extracting meaningful insights from the accumulated data. The chi-square test and Spearman correlation test were used to identify significant differences and correlations between the variables under investigation. The threshold for statistical significance was set at p<0.05, ensuring that the findings were robust and could be deemed meaningful indicators of patterns, associations, or variations within the dataset. This methodological rigor in statistical analysis not only upholds the scientific integrity of the study but also establishes a reliable foundation for deriving informed conclusions from the collected data, thereby advancing the overall objectives of the research endeavor.

## Results

As shown in Table 1, of the patients enrolled, 208 (54.0%) were male and 177 were female (46.0%); 190 (49.4%) participants fell in the age group of 20-35 years, 120 (31.2%) were aged 36-50 years, and 75 (19.5%) were over 50 years. The educational backgrounds of the participants were diverse, with 145 (37.7%) having a primary education, 123 (31.9%) secondary, and 117 (30.4%) holding a bachelor's degree. More than the half of participants (223, 57.9%) resided in rural areas, while 162 (42.1%) lived in urban settings. Regarding the duration since diabetes diagnosis, 190 (49.4%) had been diagnosed within the last one to five years, 80 (20.8%) between 5-10 years, and 115 (29.9%) for more than 10 years.

**Table 1:** Sociodemographic data and medical history of the patients (n=385)

Variables	N	%
Sex		
Male	208	54.0
Female	177	46.0
Age group, years		
20-35	190	49.4
36-50	120	31.2
More than 50	75	19.5
Education		
Primary	145	37.7
Secondary	123	31.9
Bachelor's degree	117	30.4
Residence		
Rural	223	57.9
Urban	162	42.1
Duration since diagnosis, years		
1-5	190	49.4
5-10	80	20.8
>10	115	29.9

Of note, 118 participants (30.6%) reported being "satisfied" with the current traditional diabetic management. An additional 100 (26.0%) described their satisfaction level as "average." This suggests a neutral stance among a quarter of the participants, reflecting a moderate level of contentment with the existing management approach. On the other hand, 167 (43.4%) participants reported being "unsatisfied" with the current traditional diabetic management (Table 2).

**Table 2:** Overall satisfaction level with the current traditional diabetic management among the patients (n=385)

Satisfaction level	N	%
Describe your overall satisfaction		
Satisfied	118	30.6
Average	100	26.0
Unsatisfied	167	43.4

Table 3 provides a detailed breakdown of participants' knowledge regarding artificial pancreas, assessed across various aspects. The results showed that 235 (61%) participants had a satisfactory total knowledge score while 150 (39%) had an unsatisfactory total knowledge score. The data showed that 145 patients (37.7%) provided correct and complete answers regarding the types of the artificial pancreas, followed by 97 (25.2%) giving the same about the mechanism of action, 91 (23.6%) on how it is implanted, 90 (23.4%) about the main function, and 87 (22.6%) about the main parts.

**Table 3:** Knowledge about artificial pancreas among the patients

Items	Complete and correct answer		Correct but not complete answer		Don't know	
	N	%	N	%	N	%
The main function	90	23.4	170	44.2	125	32.5
The mechanism of action	97	25.2	150	39.0	138	35.8
The main parts	87	22.6	141	36.6	157	40.8
Types of the artificial pancreas	145	37.7	120	31.2	120	31.2
How it is implanted	91	23.6	87	22.6	207	53.8
Guidelines on maintenance	54	14.0	140	36.4	191	49.6
Guidelines when traveling	67	17.4	133	34.5	185	48.1
Guidelines when exercising	41	10.6	134	34.8	200	51.9
Guidelines about disconnecting	75	19.5	140	36.4	170	44.2
Total knowledge score, n (%)						
Satisfactory	235 (61%)					
Unsatisfactory	150 (39%)					

Table 4 presents the frequency distribution of participants' attitudes toward the use of the artificial pancreas. The data showed that 247 patients (64.1%) had a positive attitude, while 138 (35.9%) had a negative attitude.

**Table 4:** Attitudes toward the use of the artificial pancreas among the patients (385)

Items		Sure (3)		May be (2)		Not sure (1)	
		N	%	N	%	N	%
1	I would be willing to try the artificial pancreas as part of my diabetes treatment	169	43.9	120	31.2	96	24.9
2	I trust the safety and reliability of the artificial pancreas technology	180	46.8	115	29.9	90	23.4
3	I believe that using the artificial pancreas would improve my overall quality of life	196	50.9	100	26.0	89	23.1
4	I feel confident in my ability to adapt to and use the artificial pancreas effectively	175	45.5	91	23.6	119	30.9
5	The potential benefits of the artificial pancreas outweigh any concerns I may have	218	56.6	72	18.7	85	22.1
6	I am optimistic about the positive impact of the artificial pancreas on diabetes treatment	223	57.9	80	20.8	82	21.3
Attitude							
Positive		247 (64.1%)					
Negative		138 (35.9%)					

Table 5 presents the frequency distribution of the studied sample concerning the most commonly perceived barriers to using the artificial pancreas. The data indicates that 145 (37.7%) participants identified the high cost as a significant barrier. Additionally, 90 (23.3%) expressed concerns about the lack of healthcare provider support, 83 (21.5%) had reservations regarding maintenance, and 67 (17.5%) felt limited by their low technical skills.

**Table 5:** Most commonly perceived barriers to using the artificial pancreas among the patients (385)

Items	N	%
High cost	145	37.7
Lack of healthcare provider support	90	23.3
Concerns regarding maintenance	83	21.5
Limited technical skills	67	17.5

Table 6 illustrates the association between sociodemographic factors and types of attitudes regarding the use of the artificial pancreas. There was a significant association between sex and attitude (X^2^=4.05, p=0.04). Females showed higher levels of acceptance compared to males. There was a highly significant association between age and attitude (X^2^=33.3, p=0.00001). Participants aged 20-35 years exhibited the highest level of acceptance. A significant association was also observed between educational levels and attitude (p=0.00001). Patients with higher education levels (Bachelor's) showed the highest acceptance levels. However, there was no significant association between area of residence and attitude (X^2^=0.052, p=0.8). Both rural and urban participants showed relatively similar acceptance rates.

**Table 6:** Association between sociodemographic data of the studied sample and their attitude toward the artificial pancreas (n=385)

Items	N	Positive (n=247)		Negative (n=138)		X^2^	P-value
		N	%	N	%		
Sex						4.05	0.04
Male	208	124	59.62	84	40.38		
Female	177	123	69.49	54	30.51		
Age group, years						33.3	0.00001
20-35	190	155	81.58	35	18.42		
36-50	120	80	66.66	40	33.34		
More than 50	75	12	16	63	84		
Education						31.01	0.00001
Primary	145	70	48.28	75	51.72		
Secondary	123	82	66.66	41	33.34		
Bachelor's degree	117	95	81.19	22	18.81		
Residence						0.052	0.8
Rural	223	142	81.19	81	18.81		
Urban	162	105	64.81	57	35.18		

As shown in Table 7, there was a highly positive correlation between the total knowledge score and acceptance of the artificial pancreas (r=0.73, p=0.000). As knowledge increases, a more positive attitude toward using the artificial pancreas is observed. There was a moderately negative correlation between age and acceptance of the artificial pancreas (r=-0.58, p=0.000). Younger individuals tended to have a more positive attitude toward using the artificial pancreas compared to older individuals. There was a moderately negative correlation between the duration from the onset of the disease and acceptance of the artificial pancreas (r=-0.61, p=0.000). Individuals with a shorter duration since the onset of the disease were more likely to have a positive attitude toward using the artificial pancreas.

**Table 7:** Correlation between knowledge score, age, and years since diagnosis among the studied sample

Items	Overall attitude	
	R	P-value
Total knowledge score	0.73	0.000
Age	-0.58	0.000
Duration since the onset	-0.61	0.000

## Discussion

This study aimed to explore the awareness, attitude, and perceived utilization barriers regarding the artificial pancreas among diabetic patients. Our findings showed that around half of the studied sample (43.4.%) expressed being "unsatisfied" with the current traditional diabetic management and about one quarter (26.0%) described their satisfaction level as "average.” This finding aligns with that of studies by Farahat et al. and Atallah et al. [13,14] while it contrasts with that of Priya et al. [15] who reported that 70.1% of the patients were satisfied with the healthcare services received. The differences between the previous studies may be attributed to the limited access and inability to control blood sugar levels.

Our results showed that 39% of the participants had an unsatisfactory total knowledge score. In the same vein, Altaib and Almehthel [9] as well as Meade and Rushton [16] reported that diabetic patients needed further education and training regarding the mechanism of action, and guidelines for using and maintenance of the artificial pancreas. The current results were supported by Alghadeer et al. [17] who found that there was a lack of knowledge among most healthcare providers about using insulin pumps and hence educational programs, professional seminars, and continuous professional education about the basic knowledge and primary principles of insulin pump therapy are needed. The current study identified that about two-thirds of the studied patients had a positive attitude toward the artificial pancreas. This finding aligns with that of van Bon et al. [11] who found that many participants (58%) had been reluctant to start continuous subcutaneous insulin infusion. Additionally, Marigliano et al. [18] reported that the patients had higher scores for acceptance toward the use of the artificial pancreas.

We found a significant association between gender and attitudes toward the use of the artificial pancreas; females showed a higher level of acceptance compared to males. There was a highly significant association between age and attitude. Participants aged 20-35 years exhibited the highest acceptance levels. A significant association was also observed between education and attitude. Higher education levels (Bachelor's) were associated with the highest acceptance rates. There was no significant association between area of residence and attitude. Both rural and urban participants showed relatively similar acceptance rates. The current is in agreement with Nuzzo and Schettino [19], who reported that females had higher acceptance levels for using the artificial pancreas than males because they have worse metabolic compensation. Regarding the effect of the educational levels, Philpott [20] revealed that people with higher education are more likely to use the artificial pancreas. The finding in this study showed that younger individuals and those with a shorter duration since the onset of the disease are more likely to have a positive attitude toward using the artificial pancreas. In the same vein, Chimento-Díaz et al. [21] concluded that younger age and a higher education level are factors associated with a greater degree of acceptance of the use of new medical technologies. In our study, younger age and shorter duration since the onset of the disease turned out to be two sides of the same coin.

Results of the present study indicated that 37.7% of participants identified the high cost as a significant barrier to using the artificial pancreas. Additionally, 23.3% expressed concerns about the lack of healthcare provider support, 21.5% had reservations regarding maintenance, and 17.5% felt limited by their low technical skills. These results are supported by Pauley et al. [22], Kapil et al. [23], and O'Donnell et al. [24]. However, van Bon et al. [11] reported that the majority of diabetic patients faced no barriers to using the artificial pancreas. The differences may be attributed to the fact that the artificial pancreas is not covered by insurance and technological support in the healthcare system in Egypt.

Limitations of the study

Although the current study provided valuable empirical data on knowledge, attitudes, and barriers to using the artificial pancreas, which can serve as a baseline for future research and interventions in similar populations or other regions, the study has several limitations. Primarily, it focused on diabetic patients in a single geographic location: Damanhur city, the Beheira Governorate, Egypt, which may limit the generalizability of the findings to other regions or diverse populations with different healthcare contexts. Moreover, while the cross-sectional design we adopted provides a snapshot of awareness, attitude, and barriers at a specific point in time, it does not capture the dynamic nature of these factors over time. The selection of cutoff scores for the total score (50%) and attitude (60%) was indeed a subjective decision. While we aimed to establish criteria that were meaningful and relevant to our study, we recognize that the choice of these thresholds introduces a level of subjectivity and could impact the generalizability of our findings.

## Conclusions

The findings of this study shed light on various aspects of diabetic patients' perspectives on the artificial pancreas. Notably, about two-thirds of the participants demonstrated a satisfactory level of knowledge, and 64.1% exhibited a positive overall attitude toward this innovative technology. However, significant barriers were identified, with 37.7% citing high costs as a major obstacle. Concerns about the lack of healthcare provider support, maintenance issues (23.3%), and perceived limitations related to technical skills (17.5%) were also highlighted. These results underscore the importance of addressing these barriers to enhance the acceptance and utilization of artificial pancreas technology among individuals with diabetes. Future interventions should focus on education, cost considerations, and support mechanisms to promote a more widespread and effective adoption of this advanced medical technology. We believe that empowering both healthcare providers and patients through ongoing educational initiatives can play a pivotal role in fostering a positive attitude and addressing concerns related to artificial pancreas technology.
